# Perception of Social Interactions for Spatially Scrambled Biological Motion

**DOI:** 10.1371/journal.pone.0112539

**Published:** 2014-11-18

**Authors:** Steven M. Thurman, Hongjing Lu

**Affiliations:** 1 Department of Psychology, UCLA, Los Angeles, California, United States of America; 2 Department of Statistics, UCLA, Los Angeles, California, United States of America; Lancaster University, United Kingdom

## Abstract

It is vitally important for humans to detect living creatures in the environment and to analyze their behavior to facilitate action understanding and high-level social inference. The current study employed naturalistic point-light animations to examine the ability of human observers to spontaneously identify and discriminate socially interactive behaviors between two human agents. Specifically, we investigated the importance of global body form, intrinsic joint movements, extrinsic whole-body movements, and critically, the congruency between intrinsic and extrinsic motions. Motion congruency is hypothesized to be particularly important because of the constraint it imposes on naturalistic action due to the inherent causal relationship between limb movements and whole body motion. Using a free response paradigm in Experiment 1, we discovered that many naïve observers (55%) spontaneously attributed animate and/or social traits to spatially-scrambled displays of interpersonal interaction. Total stimulus motion energy was strongly correlated with the likelihood that an observer would attribute animate/social traits, as opposed to physical/mechanical traits, to the scrambled dot stimuli. In Experiment 2, we found that participants could identify interactions between spatially-scrambled displays of human dance as long as congruency was maintained between intrinsic/extrinsic movements. Violating the motion congruency constraint resulted in chance discrimination performance for the spatially-scrambled displays. Finally, Experiment 3 showed that scrambled point-light dancing animations violating this constraint were also rated as significantly less interactive than animations with congruent intrinsic/extrinsic motion. These results demonstrate the importance of intrinsic/extrinsic motion congruency for biological motion analysis, and support a theoretical framework in which early visual filters help to detect animate agents in the environment based on several fundamental constraints. Only after satisfying these basic constraints could stimuli be evaluated for high-level social content. In this way, we posit that perceptual animacy may serve as a gateway to higher-level processes that support action understanding and social inference.

## Introduction

One of the most fundamental and socially important functions of the human visual system is to perceive and understand the behaviors of animate agents in the environment. When creatures move, physical laws such as gravity and inertia impose physical constraints that dictate how the body and limbs must interact with the ground surface in order to propel the creature in one direction or another. Biomechanical laws provide further constraints on the possible range of limb movements given a particular animate body structure. These laws impose universal constraints on the visual appearance of biological motion, resulting in stereotypical movement patterns and invariant visual features that are preserved across many species [1], [2]. In fact, previous research shows that human newborns [3]–[5], and even newly hatched chicks [6], [7], can distinguish between biological and non-biological motion stimuli, despite extremely limited visual experience. These findings suggest the existence of innate processes that are tuned to characteristic features of biological motion. Indeed, the propensity for detecting and analyzing biological movement patterns appears to be deeply ingrained in the brains of humans and other vertebrate species (for reviews, see [8], [9]).

In the natural environment, two types of key information can be characterized in biological motion stimuli: information about limb movements in body-centered coordinates termed as *intrinsic motion* in the present paper, and information about body motion in environmental coordinates termed as *extrinsic motion* [10]. For natural actions, limb movements (particularly the feet) are expected to be highly predictive of, and causally related to, the direction and speed of global body movement. In the present paper, we term the constraint imposed on human action due to the causal relationship between intrinsic and extrinsic motion as the motion congruency constraint. Thurman and Lu (2013) showed evidence that humans are sensitive to the congruency relation between intrinsic limb movement and extrinsic body motion. Violating the motion congruency constraint caused a significant decrease in the perception of animacy for spatially-scrambled point-light walker stimuli [11].

Although human action naturally abides by constraints imposed by physical laws, it is possible for humans to give the appearance of violating the motion congruency constraint. For instance, Michael Jackson’s moonwalk could be considered an example of violating this constraint because the lower leg movements are generally consistent with forward walking direction while the whole body, to the surprise and delight of the crowd, glides backwards on stage. Another common example of humans intentionally violating this constraint comes from sports like soccer, rugby and American football. Athletes use a variety of feinting moves that involve stutter steps and deceptive leg movements in order to bypass or avoid defenders. Feinting moves are likely effective because they momentarily decouple the predictability of global body movements from intrinsic limb movements. In fact, defenders are often trained to overcome this deception by deliberately ignoring limb movements and focusing on the opponent’s center of mass (e.g., hips).

Notably, intrinsic motion and extrinsic motion have each traditionally been studied in isolation for action perception. On the one hand, the field of biological motion perception has focused on intrinsic joint motion, using point-light stimuli that typically lack extrinsic body motion, such as the “treadmill” walker ([1], [12]–[15]; for review see [16]. On the other hand, the field of social cognition has typically focused on issues such as animacy, intentionality and social interaction using stimuli composed of simple moving shapes that exhibit extrinsic motion but lack intrinsic joint movements ([17]–[21]; for review see [22]. Consequently, research in action perception has typically been limited to elemental actions such as walking and running, while research in social cognition has instead emphasized group-level attributes such as goal-directed interactive activities among multiple agents (e.g., chasing; 18–19). While there are relevant historical and theoretical reasons for the divergence of these two fields of study, we believe there is much to be gained by synthesizing results from these two research fields and by using more naturalistic stimuli that contain both intrinsic and extrinsic motion patterns.

The current study was designed to help bridge this gap by using complex, naturalistic biological motion stimuli and examining the ability of human observers to make high-level inferences about social and interactive behaviors. The study was motivated in part by a recent study [11], in which we examined the influence of gravity, intrinsic motion, and extrinsic motion on the perception of animacy for spatially-scrambled point-light walker stimuli. The immediate visual appearance of a scrambled point-light animation is typically described as a random cloud of dots with unfamiliar and deformable shape or form. As such, these stimuli would not be expected to induce prior knowledge of human action based on body shape or familiar postures. At the same time, local information about biological kinematics is preserved in these animations. Results showed that stimuli containing both intrinsic (joint movements) and extrinsic (body translation) motion were rated as significantly more animate than less naturalistic stimuli containing only intrinsic motion (e.g., treadmill walkers). Furthermore, the results showed that three fundamental constraints must be satisfied for stimuli to be rated as highly animate: individual dot movements must be consistent with the force of gravity (gravity constraint; [1]), the intrinsic and extrinsic movements must be congruent with each other (motion congruency constraint), and the global structural organization of the stimuli must reflect the prototypical mammalian body plan (biological structure constraint).

The current experiments were designed with two specific goals. First, we sought to utilize the spatial scrambling paradigm in order to remove the influence of prior knowledge on familiar actions and to probe motion-based mechanisms contributing to high-level inference of social interaction. Although it has been shown that observers can determine simple traits such as locomotion directionality from scrambled displays [1], [23], [24], it is unclear to what extent observers can also perceive more complex social traits when human body form information has been disrupted. We showed naïve observers a series of animation sequences depicting two point-light actors engaged in various types of social interaction. Participants were asked to simply describe what they had seen on the computer screen using a free response paradigm. We applied random spatial scrambling to the point-light actors, seeking to determine whether observers would spontaneously infer animate and/or socially interactive traits in the scrambled point-light stimuli, or whether observers would instead rely on physical/mechanical descriptions in their judgments.

Secondly, focusing on complex and naturalistic biological motion stimuli (e.g. coordinated partnership in salsa dance) we sought to examine the influence of several factors on the ability to identify interactivity between agents. These factors included the presence or absence of global human body form, the presence or absence of coordinated intrinsic and extrinsic body movements and, critically, the congruency between these two sources of motion information. Specifically, based on the proposal that motion congruency may act as a fundamental constraint in perceptual animacy [11], we hypothesized that violating this constraint would have a detrimental and cascading impact on the ability to perceive interactivity between agents. This result would further highlight the importance of motion congruency for biological action perception, and would suggest a hierarchical system in which basic filters first operate to detect animate creatures in the environment based on a set of fundamental constraints [1], [11], [22]. Only after these constraints have been satisfied could higher-level processes be engaged to support action understanding and social inference.

## Experiment 1

Experiment 1 investigated the ability of naïve observers to spontaneously infer animate and/or socially-interactive traits in spatially-scrambled animations of biological motion depicting various types of social interaction. The spatial scrambling paradigm provides a useful tool for 1) eliminating the familiar appearance of human body form in point-light stimuli, 2) reducing the influence of form-based processes on judgments of animacy and interactivity, and 3) removing the dependency of responses on prior knowledge of familiar actions. Using a free response paradigm, we measured the proportion of participants who spontaneously described the stimuli in terms of animacy and/or social interactivity, as opposed to using physical and/or mechanical descriptions of the moving dot stimuli.

### Method

#### Participants

Thirty Seven Undergraduate Students (28 Female, Mean Age = 20.7±2.0 Years) Were Recruited through the Department of Psychology Subject Pool at the University of California, Los Angeles (UCLA). the Study Was Approved by the UCLA Institutional Review Board (#12-000832). They Were Given Course Credit for Participation. All Participants Had Normal Or Corrected Vision, Gave Written Informed Consent Approved by the UCLA Institutional Review Board and Were Naïve to the Purpose and Stimuli Used in the Studies.

#### Materials and procedure

All stimuli were created using Matlab (MathWorks Inc.) and the Psychophysics Toolbox [25], [26] and were displayed on a calibrated CRT monitor (60 Hz, background luminance 16.2 c/m2) powered by a Dell PC running Windows XP. Experiments were conducted in a dark room with a chin rest to maintain a constant viewing distance (35 cm).

**Figure 1:**
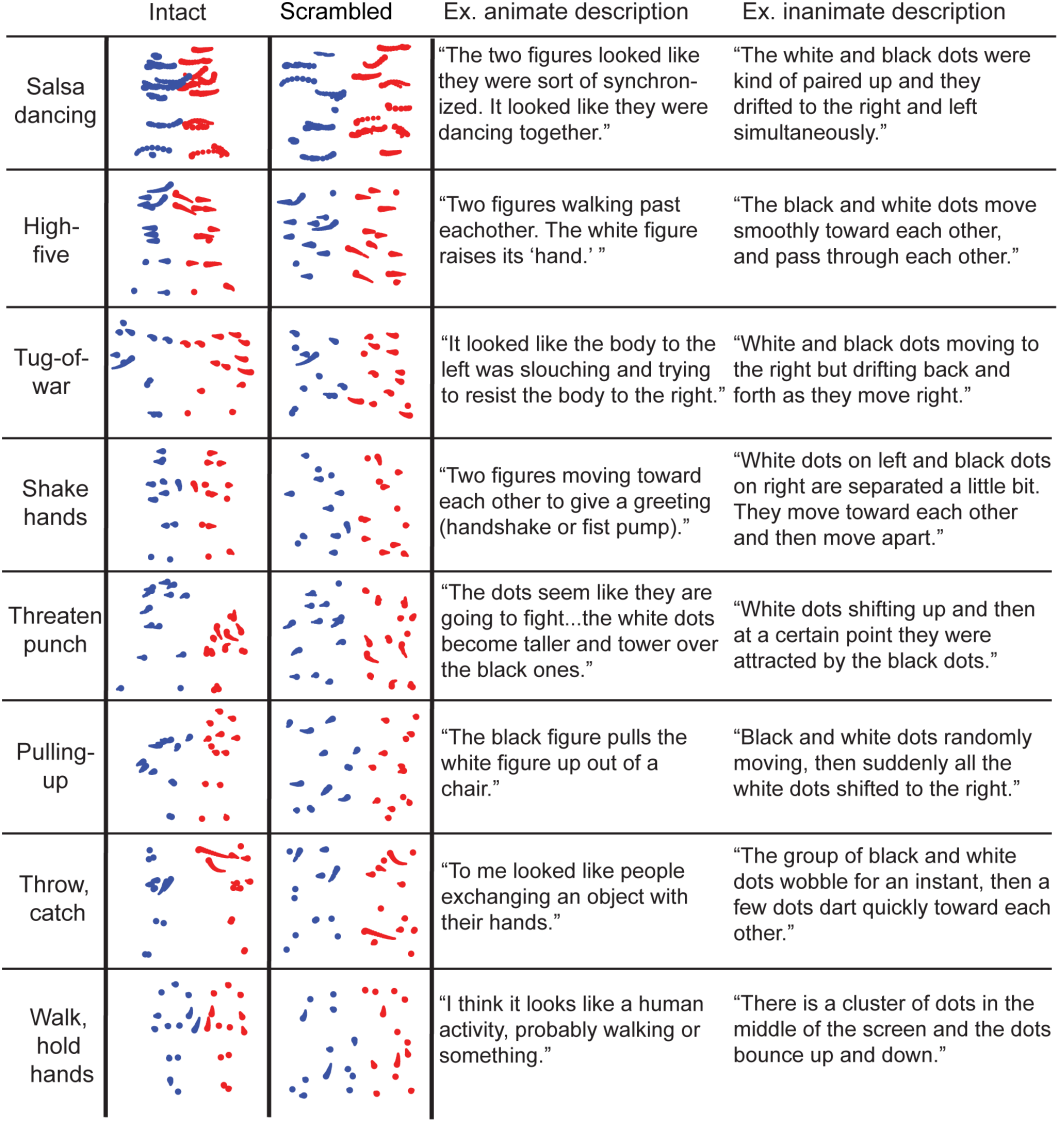
Schematic depiction of each type of interactive display (rows) with and without intact human body form, and examples of written responses. The stimuli in the first column are spatially intact for demonstration purposes, but the stimuli in the experiment were spatially scrambled versions of these animations (second column). For visualization, one dancer is colored in red dots and the other in blue dots. Dot size decreases for time points further in the past. The third and fourth columns include examples of free responses from representative participants that spontaneously used animate (third column), and inanimate (fourth column) descriptions.

The biological motion patterns of human interaction were obtained from the Carnegie Mellon Graphics Lab Motion Capture Database, available free online (http://mocap.cs.cmu.edu). Software developed in our laboratory was used to convert the raw motion capture files to point-light format, with thirteen points representing the head, shoulders, hips, elbows, wrists, knees and feet [27]. As shown in Figure 1, we tested the following eight animations of human social interaction: *salsa dancing, walking and giving a high-five, playing tug of war, walking up and shaking hands, threatening to punch while another cowers, pulling another up out of a chair, throwing and catching an object, and walking while holding and swinging hands*. These animations were chosen to represent a broad range of possible social interactions and varied in length between 3.72 and 4.33 seconds.

Each participant viewed the animations in pseudo-random order, and the order of animations was counterbalanced across participants to account for potential effects of practice and exposure. In each trial, we applied random spatial scrambling to the starting position of each point-light by choosing a random location within a rectangular region of size 5.5 deg width and height. Each dot subtended 0.39 deg in diameter. The animations were displayed on a computer screen with a gray background. The dots comprising one point-light actor were colored black, and the dots comprising the other actor were colored white, in order to facilitate grouping of the scrambled points belonging to each agent. Participants viewed each scrambled animation twice consecutively before being asked to simply describe what they had seen on the screen by writing their answers on a piece of paper. No other information was given about the supposed nature of the stimuli or the purpose of the task, and participants were urged to describe the stimuli succinctly with three sentences or less. After the experiment, subjects were asked to report whether they had any prior experience with point-light biological motion stimuli in a previous classroom or experimental setting. All of the subjects in the current experiment reported no prior knowledge of point-light biological motion stimuli.

### Results and Discussion

We coded the sentence reports participants produced to describe their perception of the scrambled moving dot stimuli. Three people (author S.M.T and two research assistants naïve to the purpose of the study) were asked to read through the responses and judge whether participants had used an animate or inanimate (physical) description. An animate description was defined as a response that contained an animate or interactive verb (e.g., walk, punch, dance, throw), and an animate noun (e.g., person, animal, creature). If a response included an animate verb but a physical noun (e.g. dot) it could still be judged as an animate response if the intent of the description was clearly construed by the judge as animate (e.g. Figure 1, see an example for *Threaten Punch*). By default, all other responses were judged as inanimate. Inanimate responses typically contained a physical noun (e.g., dots, object, figure) and a physical verb (e.g., move, rotate, slide, bounce) which described the dynamics of the display in a mechanical way. Other types of inanimate responses clearly suggest that the subject did not interpret the dynamic display in an animate or socially meaningful way (e.g. “It looked like scribbles forming a shape”). The three raters were in full agreement on the classification of responses as animate or inanimate on 89.2% of trials, demonstrating strong inter-rater consistency (mean Cohen’s *k* = 0.85, *p*<.005). Examples of participants’ descriptions satisfying these criteria are shown in Figure 1, along with a schematic depiction of each intact stimulus type.

**Figure 2:**
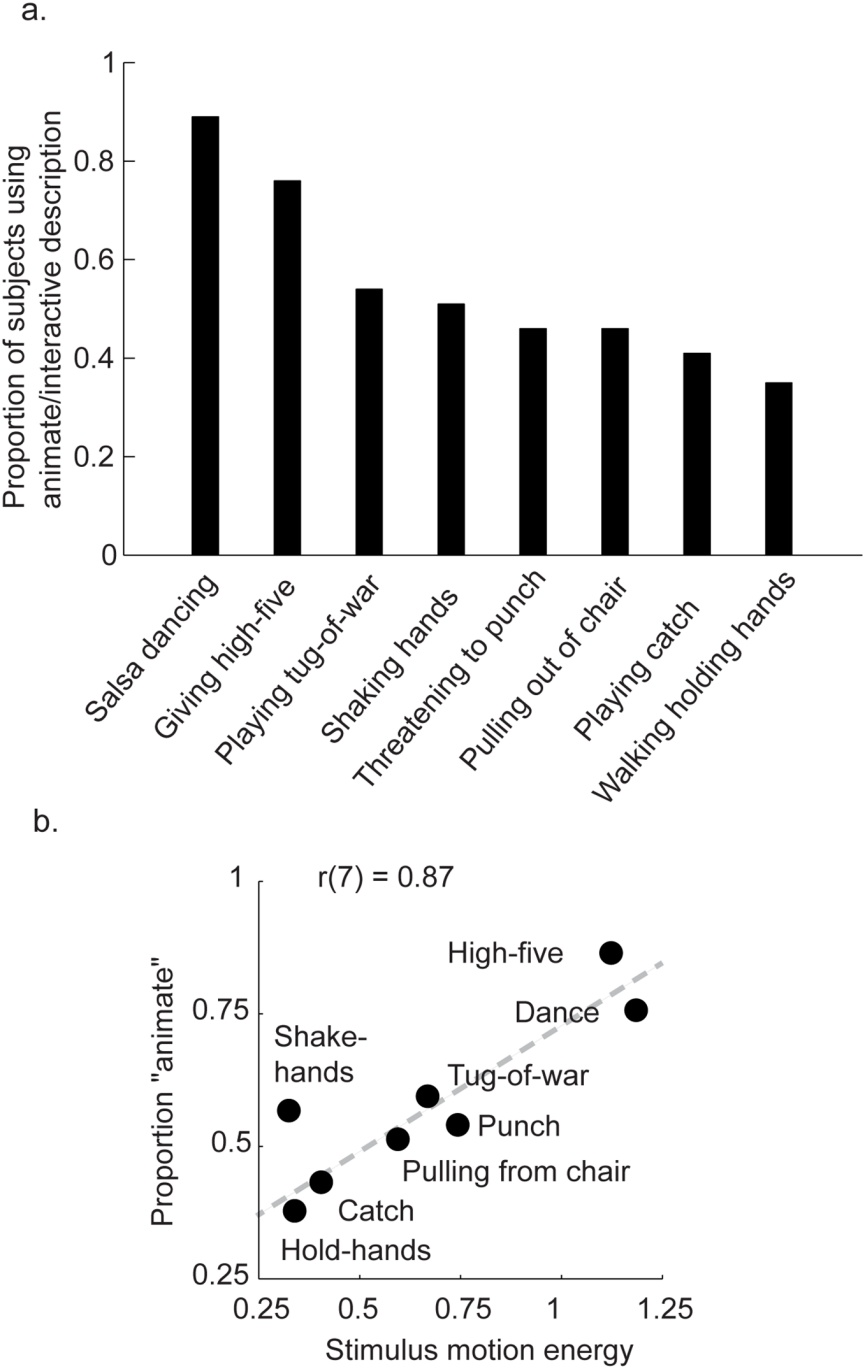
Results of Experiment 1 and analysis of motion energy contained in each display type. A) Mean data from Experiment 1 showing the proportion of subjects that spontaneously used animate/social descriptions of the spatially scrambled stimuli for each type of interactive display. B) Scatter plot with motion energy on the x-axis and likelihood of spontaneous inference of animate/social traits on the y-axis for each display type. Motion energy was computed directly from the known Euclidian displacements of the points on a frame-by-frame basis, and averaged across all points comprising both actors in the display (See Results section of Experiment 1).

Figure 2a shows the proportion of participants who used an animate description for each type of interactive display, averaged across the three independent raters. On average, 54.8% of participants attributed animate/interactive traits to spatially scrambled point-light animations. Further, we found that some interpersonal activities were more likely to signal animate/interactive traits than others. For instance, spatially scrambled salsa dancing was the most likely to be perceived as animate and interactive (89%), and walking while holding hands was the least likely to be perceived as animate and interactive (35%). To account for the differences among various interpersonal activities, we hypothesized that some low-level features of body movement in the scrambled point-light displays might contribute to the perception of animate/socially-interactive traits. The pattern of data in Figure 2a makes it apparent that displays perceived as highly animate/interactive (e.g., *salsa dancing, walking and giving high-five, playing tug-of-war*) were those that had the greatest amount of body movement over time. We evaluated the total amount of motion energy available from both actors in each display over the course of the entire interaction sequence. This was computed as the sum of the averaged inter-frame Euclidian displacement of all dots in the display, and then normalized by the total number of frames in the sequence. Indeed, we found that the pattern of total motion energy across the interactive displays (Figure 2b) was highly correlated with behavioral data, *r*(7) = 0.87, *p* = 0.006. This result suggests that in general, within the range of natural biological movements between two agents, scrambled displays with faster joint movements and more pronounced global body motion tend to evoke a stronger sense of animacy and interactivity than displays with weaker motion signals.

## Experiment 2

The results of Experiment 1 make two important points. First, naïve observers can spontaneously recognize animacy and social interaction in spatially scrambled point-light displays that lack information about human body form. This suggests that dynamic properties of the stimulus were sufficient to evoke prior knowledge of biological action and interaction, independent of the form processing system. Secondly, the degree of animacy and interactivity that observers could perceive in different action animations was affected, at least in part, by the total amount of motion energy present in the stimulus.

In Experiment 2, we sought to further examine the ability of human observers to perceive social interaction using a more rigorous psychophysical discrimination task. We employed several different animations of human salsa dancing and asked observers to discriminate which of two intervals contained partnered, interacting dancers, where the distracter interval contained de-synchronized and un-partnered salsa dancers. We chose to focus on salsa dance for three reasons. First, the results of Experiment 1 showed that observers were most likely to spontaneously attribute animate and interactive traits in scrambled displays of salsa dance as compared to all other interpersonal activities tested. Second, a rich set of motion capture data of human salsa dancing was readily available online and was sufficiently long to break the sequence into several shorter snippets for use in the psychophysical experiment. Third, meaningful interaction in human salsa dance is characterized by a combination of coordinated limb movements (e.g., hands of each partner touching during a spin move), as well as coordinated whole-body movements (e.g., when one actor steps forward the other steps back, etc.) between the two partnered dancers. Hence, we expected that intrinsic motion and extrinsic motion should each provide useful information for recognizing and understanding interaction in the salsa dance displays.

Several experimental conditions were included in Experiment 2. First, the stimuli were either spatially intact or spatially scrambled in order to measure performance levels with intact human body form and without global form information. Previous studies have shown that observers can accurately discriminate locomotion direction from scrambled displays [1], [24], [28], [29], and we sought to determine if observers could also identify complex social traits such as meaningful interaction from scrambled stimuli. Next, we manipulated the stimuli so that the coordinated movements between the salsa dancers would be preserved for both intrinsic and extrinsic motion (i.e., normal partnered sequence), intrinsic motion only (i.e., partnered limb movements, but unrelated global body motion between agents), extrinsic motion only (i.e., partnered global body motion, but unrelated limb movements of the two agents), or for neither intrinsic nor extrinsic motion (i.e., completely un-partnered). We analyzed the varying contributions of each of these form and motion-based visual cues to discrimination performance, expecting that performance would decrease as a result of both spatial scrambling and decoupling the congruency between intrinsic and extrinsic motion.

### Method

#### Participants

Thirty undergraduates (23 female, mean age = 19.9±1.3 years) were recruited through the UCLA Department of Psychology subject pool and given course credit for participation. The study was approved by the UCLA Institutional Review Board (#12-000832). All participants had normal or corrected vision, gave written informed consent approved by the UCLA Institutional Review Board and were naïve to the purpose and stimuli used in the studies. A total of 5 participants failed to achieve criterion level performance of at least 60% correct (chance level = 50%) with spatially-intact stimuli in the training session, and were excluded from participation in the main experiment.

### Materials and procedure

The experimental setup was similar to Experiment 1. We obtained various animations of paired human salsa dancers from the Carnegie Mellon Graphics Lab Motion Capture Database, available free online (http://mocap.cs.cmu.edu). We acquired ten continuous salsa dance sequences that totaled over 135 sec in length, and divided the sequence into 27 unique snippets totaling 5 sec each. Thus, each 5 sec animation contained motion capture data of two human partners engaged in meaningful interaction through salsa dance.

Stimuli were presented on a computer monitor (60 Hz) with gray background, while the dots comprising one actor were colored white and the dots of the second actor were colored black. This was done to facilitate grouping of points belonging to each actor, which was particularly important for the conditions with spatially scrambled stimuli due to the lack of familiar form cues to help organize the dots. Each dot subtended 0.39 deg in diameter, and each actor subtended on average 5.5 deg in height.

**Figure 3:**
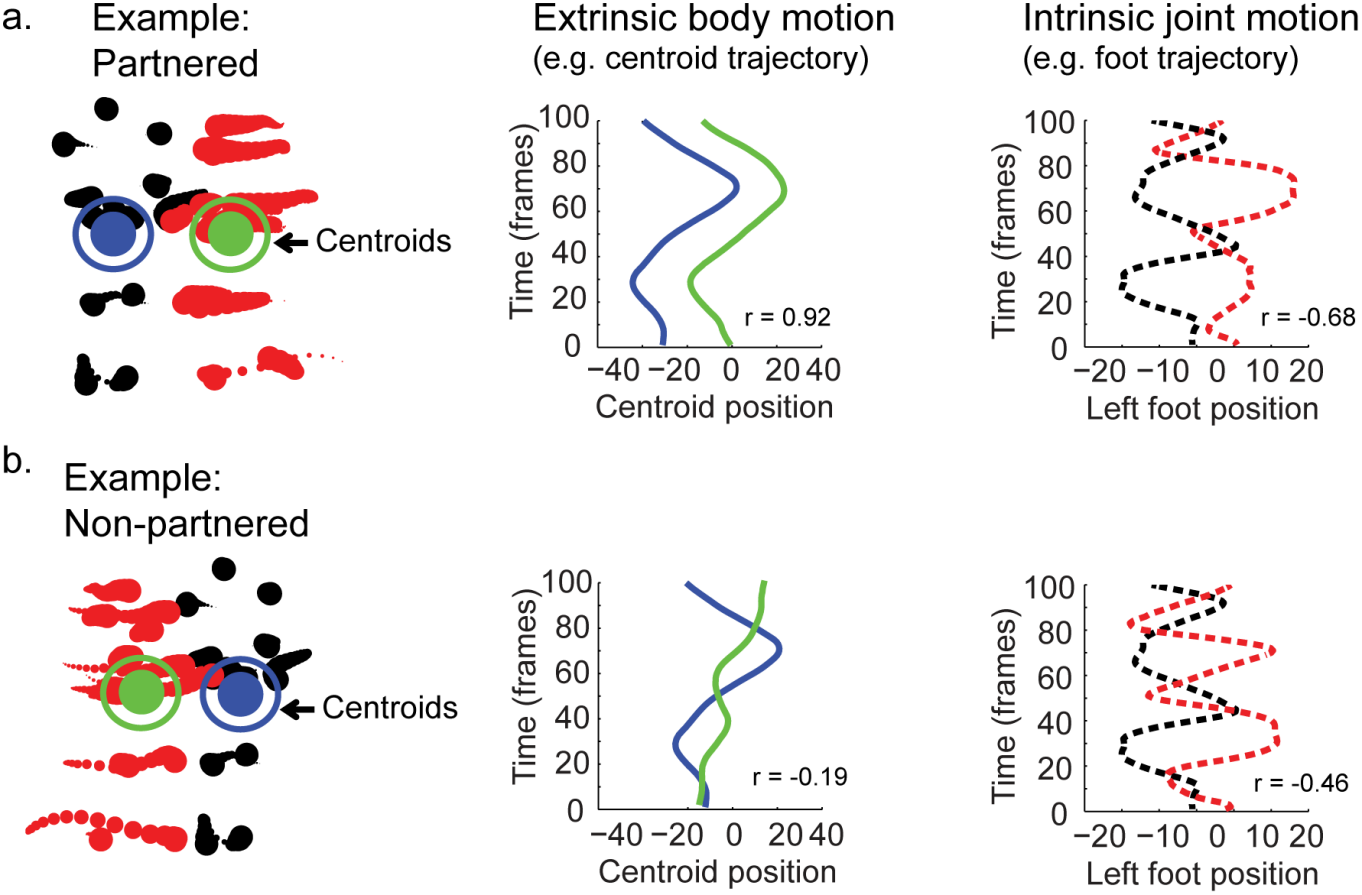
Schematic depiction of sample stimuli with plots showing motion profiles of extrinsic and intrinsic motion. (a) Left: Example of truly-partnered dancers. For visualization, one dancer is colored in red dots and the other in black dots. Dot size decreases for time points further in the past. Blue and green target symbols indicate roughly the centroid of the figure. Middle: Position of the centroid of each figure over time (in pixels), illustrating graphically the profile of extrinsic motion. Right: Position of the left foot point from each figure over time (in pixels) after subtracting extrinsic motion, illustrating the profile of intrinsic motion for an example joint. (b) Same as above, but for un-partnered dancers taken from two different “snippets”. Notice there is a stronger inherent correlation (Pearson’s *r*) between the intrinsic and extrinsic movements of dancers that are truly-partnered versus un-partnered.

Before the main experiment commenced, participants completed a short block of 24 practice trials in order to gain familiarity with the task and the salsa dancing stimuli. On each practice trial, participants viewed two intervals, each containing a different pair of salsa dancers with intact body form (not scrambled). One interval was randomly designated the target interval and contained truly partnered salsa dancers from the same action snippet (Figure 3a). The distracter interval contained two salsa dancers each chosen randomly from different, un-partnered snippets out of the total collection of 27 snippets (Figure 3b). Although the distracter interval contained two dancers in close spatial proximity, unlike the target interval, their body movements and actions were uncoordinated over time due to random selection from different snippets. Subjects performed a 2-interval forced choice task (2AFC) discriminating partnered versus un-partnered activity. If their performance reached criterion level (60%), then the participants continued with the main experiment. On average, subject accuracy on the practice block was 88% (*SD* = ±12).

The main experiment had a 2×2×2 within-subjects design. The first factor was spatial scrambling in which target and distracter stimuli were either spatially intact or spatially scrambled. When scrambled, we randomized the starting position of each point comprising an actor within a square region of 5.5 deg height and width. The second factor was intrinsic motion, either partnered or non- partnered between the two actors in the target interval. The third factor was extrinsic motion, either partnered or non-partnered between the actors in the target interval. Subjects performed a temporal two-alternative forced-choice task, identifying the target interval with interacting actors. Every trial had a distracter interval similar to the training block, in which two salsa dancers were randomly chosen from two different snippets, thereby rendering their body movements uncoordinated and temporally out of phase.

We introduced the following alterations to the target point-light stimuli in order to manipulate intrinsic and extrinsic motion independently [10]. First, we measured the component of extrinsic motion by tracking the centroid of each actor (mean position of all body points) over time. Next, we subtracted this component on a frame-by-frame basis from the original sequence to create a new animation that would essentially dance “in-place” without extrinsic body movements (see Figure 3), similar to the method for creating *treadmill* walkers in previous studies. On trials with partnered intrinsic motion, we maintained the coordinated intrinsic movements between actors by using actors from within the same snippet. When intrinsic motion was non-partnered, we used intrinsic movements derived from two actors chosen randomly from different snippets. On trials with partnered extrinsic motion, we maintained the coordinated extrinsic body movements between actors by incorporating the extrinsic motion of actors from within the same dancing snippet. Likewise, extrinsic motion was made to be non-partnered by incorporating extrinsic movements from dancers from two different snippets. Extrinsic motion was incorporated by simply adding a particular component of extrinsic motion back into the intrinsic-only motion stimuli on a frame by frame basis. Importantly, when only intrinsic or extrinsic motion was partnered, and the other type of motion was non-partnered, this created a situation in which intrinsic movements were decoupled from extrinsic movements, thereby violating the aforementioned motion congruency constraint on biological movement.

All conditions were balanced and randomly intermixed within two blocks of 72 trials, totaling 18 trials per condition. Subjects were informed that some trials would consist of spatially scrambled animations that would not appear human-like appearance, but would nonetheless retain joint movements consistent with human salsa dancing. Subjects responded by pressing keys 1 or 2 on the keyboard to indicate which interval was perceived to have more interactivity and partnership between the two point-light figures.

### Results and Discussion

**Figure 4:**
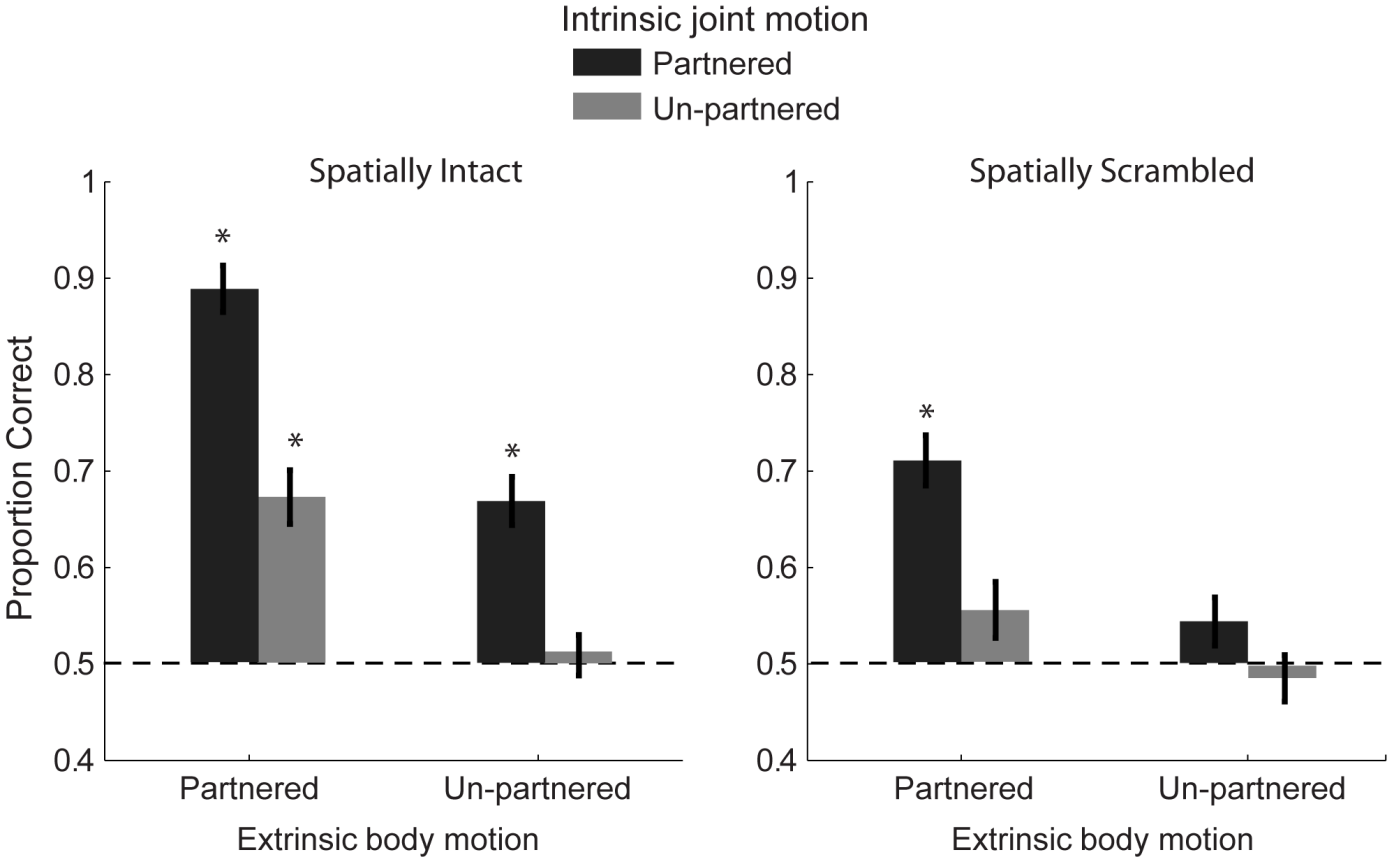
Mean group data from Experiment 2, showing the proportion of correct trials discriminating partnered from non-partnered salsa dancers for spatially intact (left) and spatially scrambled (right) stimuli. The dotted line represents chance level performance, and the asterisks indicate performance significantly greater than chance (one-sample *t-tests*). Error bars represent SEM.

We computed the proportion of correct trials for each condition, and mean performance is presented in Figure 4. To evaluate statistical differences among these conditions, we conducted a 2×2×2 within-subjects analysis of variance. For spatially-scrambled stimuli that lacked human body form, participants could achieve above chance performance as long as intrinsic and extrinsic motion were both matching and congruent with each other (accuracy of 0.71±0.026, *t*(24) = 8.16, *p*<0.001). This demonstrates that there was sufficient useful information in the patterns of motion trajectories, independent from global form, to determine the interactivity between scrambled biological agents. However, when stimuli were scrambled and the congruency between intrinsic and extrinsic motion was violated (e.g. non-matching extrinsic motion, accuracy of 0.56±0.029, or non-matching intrinsic motion, accuracy of 0.54±0.025), subjects could not perform the task above chance level (one-sample *t*-tests, both *p*’s>0.05).

Compared to spatially scrambled stimuli, performance was significantly increased for the intact condition in the presence of human body form, *F*(1,24) = 43.8, *p*<0.001, *η_p_* ^2^ = 0.65. Furthermore, with the availability of intact human form, observers showed some tolerance to incongruency between intrinsic and extrinsic motion cues, with performance significantly above chance level when either intrinsic motion was matching, accuracy 0.67±0.028, *t*(24) = 6.1, *p*<0.001, or extrinsic motion was matching, accuracy 0.67±0.025, *t*(24) = 6.8, *p*<0.001, while the other type of motion cue was non-matching. However, observers only appeared to have access to these cues when global form was intact, supporting the hypothesis that the availability of global body form information may provide a reference frame to facilitate efficient processing of the biological motion information [30]. This interpretation is supported by the results of the ANOVA, which revealed a significant interaction effect between scrambling and intrinsic motion matching, *F*(1,24) = 5.6, *p* = 0.027, *η_p_* ^2^ = 0.19, and between scrambling and extrinsic motion matching, *F*(1,24) = 5.3, *p* = 0.031, *η_p_* ^2^ = 0.18. Together, these results potentially reveal a fundamental property of the visual system: violation of naturalistic constraints such as motion congruency and biological structure not only reduces perceptual animacy [11], but also appears to effectively disable or diminish the processing of high-level social traits in biological motion stimuli.

## Experiment 3

The aim of Experiment 3 was to further explore the ability of human observers to determine social interactivity in scrambled salsa dance displays. Participants were asked to rate spatially scrambled animations of salsa dance on a scale of 1 to 5 in terms of the perceived interactivity between the actors. The rating task provided two important benefits to complement the two interval forced-choice discrimination task employed in Experiment 2. First, because there was no strict definition of chance level performance, the subjective rating task would potentially serve as a more sensitive measure for differences among spatially scrambled stimuli in terms of the congruency between intrinsic and extrinsic motions. This is important because of the possibility of a statistical *floor effect* contributing to poor, chance-level performance in some of the conditions when the point-light actors were spatially scrambled in Experiment 2. Second, we expected that subjective interactivity ratings should reflect the natural inclination of participants to perceive coordinated partnership in each type of scrambled display. Thus, instead of making a comparative judgment relative to a specific distracter stimulus, the rating task in Experiment 3 aimed to measure the degree to which changes in intrinsic and extrinsic motion congruency would directly affect the subjective impression of interactive activities between scrambled agents.

### Method

#### Participants

Twenty participants (13 female, mean age = 20.5±1.8 years) were recruited through the UCLA Department of Psychology subject pool and given course credit for participation. The study was approved by the UCLA Institutional Review Board (#12-000832). All participants had normal or corrected vision, gave written informed consent approved by the UCLA Institutional Review Board and were naïve to the purpose and stimuli used in the studies.

#### Materials and procedure

The experimental setup and salsa dancing stimuli were analogous to those used in the spatially scrambled conditions in Experiment 2. Before the main experiment commenced, participants performed the same practice block of 24 trials discriminating partnered from non-partnered salsa dancers that were spatially intact. On average, subject accuracy on the practice block was 89% (*SD* = ±12). The purpose of this was to familiarize subjects with the point-light displays and to make explicit what was meant by “interactivity” (i.e., coordinated partnership) in human actions. The main experimental task and procedure differed from Experiment 2 in the following ways. Every trial contained two groups of dots generated from spatially scrambled point-light salsa dancers, either from a pair of truly-partnered dancers or from un-partnered dancers taken from different random snippets. Participants were asked to subjectively rate on a scale of 1(least) to 5 (most), how interactive or partnered the displays appeared on each trial. Prior to the task, participants were informed about the nature of the spatial scrambling procedure and were asked to try to use the same definition of interactivity that was learned during the practice block, despite the loss of global body form information in the scrambled displays.

To manipulate the congruency of intrinsic and extrinsic motion of each actor, we employed the same general technique as used in Experiment 2. First we isolated the component of intrinsic motion for each point in body-centered coordinates by subtracting the common extrinsic motion component on a frame by frame basis. Next, we reversed the time course of extrinsic motion for each actor so that global body movements would occur in reverse temporal order as compared to the normal, forward temporal order of intrinsic limb movements. Notably, this procedure ensured that intrinsic movements would no longer be predictive of the direction of extrinsic body movement. However, this procedure also ensured that the correlated, and coordinated, nature of extrinsic body movements between partnered salsa dancers would be maintained over time. That is, intrinsic motion and extrinsic motion would each individually have the same inherent correlation between the actors in both congruent and incongruent displays. The key difference was that the motion congruency constraint would be violated in the incongruent displays due to the inconsistency between intrinsic and extrinsic motion cues.

All trial types were balanced and randomly intermixed within 2 blocks of 80 trials, totaling 40 trials per condition. Responses were recorded on a keyboard using the arrow keys to toggle among the 5 rating options, and the enter key to record the response. Each trial lasted a total of 5 seconds and responses were self-paced. Participants were asked to try their best to utilize the entire range of responses from 1 to 5 across trials.

### Results and Discussion

**Figure 5:**
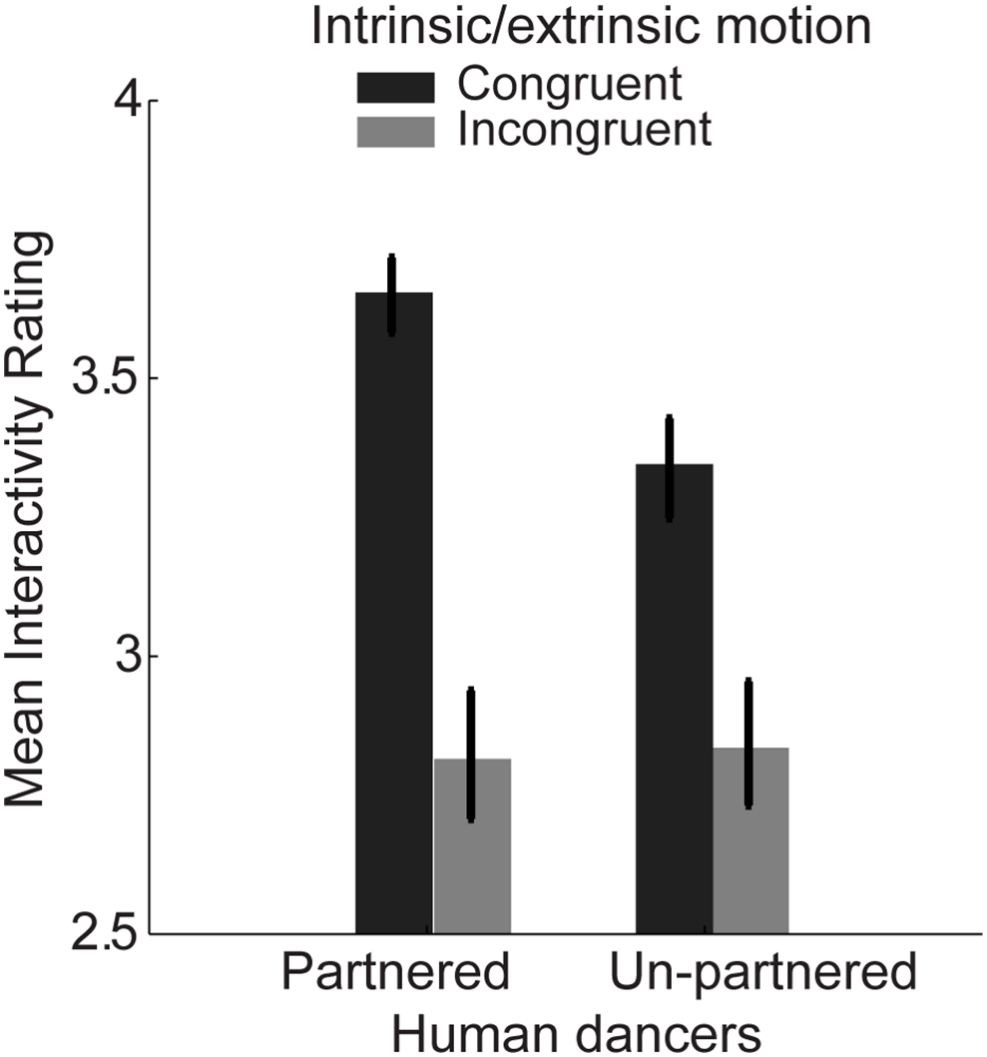
Mean group data from Experiment 3, showing mean interactivity ratings (on a five point scale; higher means more interactive) for spatially-scrambled salsa dancers. Salsa dancers were either paired with their true partner or a dancer chosen randomly from a different “snippet”. Intrinsic/extrinsic motion congruency was manipulated by reversing the timecourse of extrinsic motion for each actor in the display, maintaining the overall coordination between the actors in terms intrinsic and extrinsic motion individually, but violating the consistency between these two sources of motion information. Error bars represent SEM.

For each participant, we computed the mean interactivity rating across all trials for each condition. Mean group data is displayed in Figure 5. A 2×2 within-subjects analysis of variance revealed that ratings were significantly higher, on average, for partnered vs. non-partnered displays, *F*(1,19) = 17.9, *p*<0.001, *η_p_* ^2^ = 0.49. This result supports the idea that subject ratings indeed reflected the degree of perceived interaction among the scrambled figures, and not motion congruency or animacy per se. Ratings were also significantly higher for congruent versus incongruent motion displays, *F*(1,19) = 73.1, *p*<0.001, *η_p_* ^2^ = 0.79, suggesting observers were sensitive to the congruency between intrinsic limb movements and extrinsic body motion, which in turn affected their judgment on interactivity. We also found a significant interaction effect between these two factors, *F*(1,19) = 21.5, *p*<0.001, *η_p_* ^2^ = 0.53. The degree of true partnership between two actors contributed significantly to interactivity ratings only when point-light animations maintained congruency between intrinsic and extrinsic motion, with mean rating in the partnered condition as 3.65±0.07, and mean rating in the un-partnered condition as 3.3±0.09, *t*(19) = 4.8, *p*<0.001, but not for incongruent stimuli, with mean rating in the partnered condition as 2.82±0.12, and mean rating in the un-partnered condition as 2.84±0.11, *t*(19) = −0.73, *p* = 0.476. That is, when intrinsic limb movements were incongruent with extrinsic body motion, observers apparently lost sensitivity to the information signaling meaningful interaction through coordinated joint and body movements. Interestingly, interactivity ratings were actually higher for un-partnered dancers that maintained motion congruency, with mean rating of 3.38±0.067, as compared to the ratings for truly-partnered dancers that happened to violate the motion congruency constraint with mean rating of 2.82±0.12, *t*(19) = 6.8, *p*<0.001. Together, these results indicate that observers had general difficulty in perceiving meaningful interaction between two agents if body form information was eliminated and the expected congruency between intrinsic and extrinsic motion was also violated.

## General Discussion

The purpose of the current study was to investigate the ability of human observers to perceive and discriminate social and animate traits in complex displays of human biological motion. Few previous studies in biological motion have focused directly on perception of meaningful interaction among multiple agents. Some studies have used interacting point-light stimuli (e.g., fighting and dancing) to show that meaningful interaction can facilitate detection and discrimination of human actions embedded in noise dots [31], [32]. Interacting point-light stimuli have been used to probe form-based processing of biological motion at global and intermediate levels of structural analysis, showing that observers can utilize limb-based representations to process human actions [33]. One recent study directly assessed the specific visual cues contributing to the perception of meaningful interaction in multi-agent displays [34]. Results revealed view dependencies in the perception of social interaction. A specific combination of visual cues including joint velocities, opponent joint movements, and correlated joint velocities among interacting agents, could help explain performance for each interaction type [34]. In the context of the current study, these latest findings are important in showing that the ability to explicitly recognize social content in multi-agent displays depends strongly on the presence of relevant motion features and spatio-temporal properties [35].

Across three experiments, we examined the contribution of form and motion-based cues to the recognition of human interaction using a variety of experimental methods. Experiment 1 focused on spontaneous recognition of animate and socially interactive traits in spatially scrambled displays of human social interaction. Although observers were unable to explicitly recognize human body shape in the scrambled display, observers still attributed animate and/or social traits to some scrambled stimuli. We also found that differential rates of inferred animacy across the various social animations were correlated strongly with the total amount of local and global body motion. Specifically, scrambled animations characterized by high amounts of body motion (e.g., *salsa dancing*) were spontaneously recognized as animate and socially interactive more often than animations with less body motion (e.g., *throwing and catching an object*).

The results of Experiment 2 showed that most observers could accurately recognize interaction and partnership in salsa dance animations with relatively little training, and that global form information could help to facilitate this processing. It is unclear why a small subset of participants (5 out of 30) was unable to perform the interaction discrimination task at criterion level during training, despite relatively high performance for the remaining set of participants. We suppose that these participants may have had trouble understanding the task or knowing which cues to attend to discriminate dancing interactions accurately (e.g. perhaps due to less familiarity or experience with salsa dancing). Notably, previous studies have documented significant individual differences in terms of biological motion perception [35]–[37], action adaptability [38], and fMRI brain responses to biological motion [39]. Understanding why some participants may have inherent difficulty perceiving social interactions could provide an interesting avenue of research for future studies of individual differences in biological motion perception in both normal and clinical populations [40].

The results of Experiment 2 showed that with body form intact, observers could use partnered intrinsic joint movements or partnered extrinsic body movements to discriminate social interactions above chance level. This result emphasizes the independence of these two types of motion in terms of visual processing [10]. For instance, observers could still perform the task if the only signal to interaction was carried by coordinated joint movements, despite the fact that extrinsic movements were derived from uncoordinated and un-partnered dancers. However, this was the case for only spatially intact displays, and this ability appeared to break down for scrambled displays. Performance only reached significantly above chance level with congruent and fully-partnered pairs of dancers in the spatially scrambled condition. It seems that form information served to both invoke prior knowledge of animacy based on human body structure [11], and to provide an efficient reference frame for processing and integrating motion information across the body joints [30], [41]. This interpretation is consistent with prior findings [11], and suggests that motion congruency may serve as a fundamental constraint in both detecting animate agents and in understanding the high-level social content embedded in paired biological actions.

**Figure 6:**
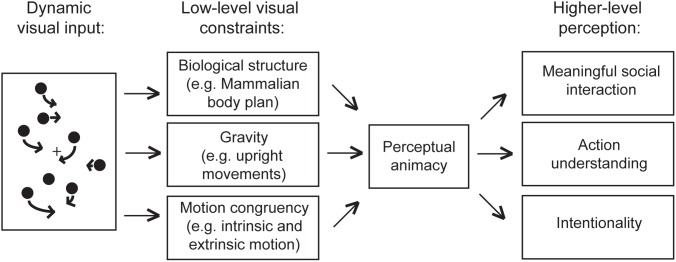
Schematic diagram of theoretical framework. When the visual system experiences a dynamic stimulus, its spatio-temporal properties are analyzed to confirm their consistency with basic constraints such as biological form, the influence of gravity, and the causal relationship between intrinsic limb movements and extrinsic body movements. These processes are relatively low-level and likely automatic or reflexive [22]. If the stimulus satisfies these constraints, it is perceived as animate and may pass on to higher-level analysis to evaluate its social significance.

Prior research on perceptual animacy [22] and biological motion suggests that animacy may be initially determined by direct, low-level processes that analyze the consistency between spatio-temporal properties of a given stimulus and several fundamental constraints on biological activity (e.g., gravity, motion congruency, body shape or form). Based on results of the current study, we speculate that animacy may serve as an effective gateway to social processing. In this theoretical framework, only if these basic constraints are satisfied can stimuli pass through to higher-level processing where social inference and action understanding can be most accurately achieved (see Figure 6).

The results of Experiment 3 also appear consistent with this proposal. The task of participants was to rate the degree to which scrambled salsa dance stimuli appeared to be interacting, or dancing as coordinated partners. The fact that there was a main effect of interactivity for truly partnered vs. un-partnered displays does suggest that these interactivity ratings reflected the degree of perceived interaction between the scrambled salsa dancers, and not motion congruency per se. Perhaps the most interesting result of Experiment 3 was that observers could only perceive differences in interactivity between truly-partnered and un-partnered displays if congruency was maintained between intrinsic and extrinsic motion. In other words, social interaction became practically imperceptible to observers when motion congruency was violated and global form was removed.

In Experiment 3, we chose to employ a subjective rating task on social interactivity as a way of complementing the results of the interaction discrimination task in Experiment 2. We acknowledge that subjective rating tasks, on their own, can have certain limitations on making inferences without a strong theoretical framework or other supporting evidence. However, two-alternative forced choice tasks also suffer from limitations due to the fact that the experimenter much choose the control stimulus to be compared to the test stimulus. In our case, the control stimuli were random pairs of dancers that were out of phase by at least 5 seconds, whereas the test stimuli were pairs of dancers that were interacting (in-phase) to various degrees depending on the partnership of intrinsic and/or extrinsic motion information. In any discrimination task, results will be conditional to some degree on the choice of control stimulus. We believe that the subjective interactivity rating task has the benefit that observers will not have to make comparative judgments between test and control stimuli, and that there is no strict definition of chance or floor-level performance. Rather, we expect the rating task to be based directly on the natural dimensions of the point-light stimuli shown on each trial. Subjective rating tasks have a long history of use in the social sciences and in cognitive psychology in particular. For example, in a related study we used reverse correlation to recover the shape of internal templates, which resembled the prototypical mammalian body plan, using subjective ratings of animacy on scrambled human point-light walkers [11]. In fact, rating tasks are especially common in studies of perceptual animacy and causality [22].

In summary, the results of Experiment 1 established that many naïve observers could spontaneously identify animate and social traits in scrambled biological motion stimuli using a free response paradigm in which participants were not informed about potential biological or social content within the moving dot animations. Experiments 2 and 3 further demonstrated that violating the motion congruency constraint in human action appears to directly inhibit the ability to extract meaningful social content from displays of human interaction. It has been theorized that early visual filters exist that are tuned to characteristic spatio-temporal properties of animate objects in the environment. We speculate that visual stimuli that fail to pass through these filters as a result of violating fundamental constraints such as gravity, motion congruency, structural appearance [1], [11], and perhaps others, may subsequently fail to take on the subjective qualities of living things including the capacity for intentional action and meaningful social interaction. In fact, a similar framework has been theorized to underlie the ability of human infants to extract meaningful social information from the environment, despite significant limitations to working memory capacity and social experience [42]. It is argued that since animacy detection and identification appear to arise quite early in human development, these more basic processes could serve as the foundation for later development of more complex social skills such as joint attention, intentionality and mutual interaction. Further studies are needed to fully characterize the spatio-temporal tuning properties of these hypothesized “animacy detectors” and their developmental origin, and to better understand the precise relationship between perceptual animacy and higher-level social processing.
